# Draft Genome Sequences of Strains Representing Each of the *Elizabethkingia* Genomospecies Previously Determined by DNA-DNA Hybridization

**DOI:** 10.1128/genomeA.00045-16

**Published:** 2016-03-10

**Authors:** Ainsley C. Nicholson, Ben W. Humrighouse, James C. Graziano, Brian Emery, John R. McQuiston

**Affiliations:** Special Bacteriology Reference Laboratory, Bacterial Special Pathogens Branch, Division of High-Consequence Pathogens and Pathology, Centers for Disease Control and Prevention, Atlanta, Georgia, USA

## Abstract

Draft genome sequences of *Elizabethkingia meningoseptica* and representatives of each of its four historically described genomospecies were sequenced here. Preliminary analysis suggests that *Elizabethkingia miricola* belongs to genomospecies 2, and both *Elizabethkingia anophelis* and *Elizabethkingia endophytica* are most similar to genomospecies 1.

## GENOME ANNOUNCEMENT

Patients infected with various *Elizabethkingia* strains have a high mortality rate, with reports of 25% for adults undergoing dialysis (1) and up to 57% for neonates with meningitis (2). Isolates from this genus are phenotypically very similar (3) but can be separated into five groups distinguished by DNA-DNA hybridization (4, 5). The type species *Elizabethkingia meningoseptica* (labeled here as KC1913) was transferred from *Chryseobacterium* to the *Elizabethkingia* genus in 2005, when the second species of the genus, *Elizabethkingia miricola*, was published (6). The third species, *Elizabethkingia anophelis*, was described in 2011 (7), and the fourth, *Elizabethkingia endophytica*, was described in 2015 (8). However, no comparisons have been made to date between the latter three species and the original genomospecies determined by DNA-DNA hybridization.

Each of the five *Elizabethkingia* isolates that was used previously as the reference genome for DNA-DNA hybridization experiments (5) was grown on heart infusion agar supplemented with 5% rabbit blood agar (RBA) at 35°C. DNA was extracted using the Joint Genome Institute (JGI) bacterial DNA isolation cetyltrimethylammonium bromide (CTAB) protocol (9). Libraries were prepared using the TruSeq DNA sample prep kit, according to the manufacturer’s instructions, and sequence reads were generated using the Illumina MiSeq instrument (Illumina, Inc., San Diego, CA). Assemblies were prepared using the CLC Genomics Workbench version 7.51 assembler (CLC bio, Waltham, MA), with automated settings using reads that were trimmed for quality (limit, 0.05%) and had adapters removed and then mapped back to contigs. Low-coverage contigs and contigs <500 bp were excluded. Sequence reads from the type strain of *E. miricola* were downloaded from the GenBank Sequence Read Archive (accession no. DRR016064) and assembled similarly.

16S rRNA gene sequences were extracted from each of the assemblies, aligned with gene sequences for the type strains of *E. meningoseptica, E. anophelis*, and *E. endophytica* that were in the public domain, and a neighbor-joining tree was generated (data not shown). While a comprehensive comparison of *Elizabethkingia* genomes is forthcoming, this preliminary analysis suggested that *E. miricola* is most similar to genomospecies 2, while both *E. anophelis* and *E. endophytica* are most similar to genomospecies 1. The 16S rRNA sequence from the recently published draft genome of *Elizabethkingia* strain ATCC 33958 (10) was an exact match to the gene from *Elizabethkingia* genomospecies 3.

### Nucleotide sequence accession numbers.

The complete genome sequences have been deposited at GenBank under BioProject no. PRJNA301708. The accession and BioSample numbers for each strain are shown in Table 1.

**TABLE 1:** BioSample and accession numbers for each *Elizabethkingia* strain

Strain	*Elizabethkingia* organism	BioSample no.	Accession no.
KC1913	*E. meningoseptica*	SAMN04254555	LNOH00000000
0422	*Elizabethkingia* genomospecies 1	SAMN04254539	LNOG00000000
G4071	*Elizabethkingia* genomospecies 2	SAMN04254557	LNOI00000000
G4075	*Elizabethkingia* genomospecies 3	SAMN04254558	LNOJ00000000
G4122	*Elizabethkingia* genomospecies 4	SAMN04254563	LNOK00000000
